# On the Large Near-Field Enhancement on Nanocolumnar Gold Substrates

**DOI:** 10.1038/s41598-019-50392-w

**Published:** 2019-09-26

**Authors:** Pablo Díaz-Núñez, José Miguel García-Martín, María Ujué González, Raquel González-Arrabal, Antonio Rivera, Pablo Alonso-González, Javier Martín-Sánchez, Javier Taboada-Gutiérrez, Guillermo González-Rubio, Andrés Guerrero-Martínez, Luis Bañares, Ovidio Peña-Rodríguez

**Affiliations:** 1 Instituto de Fusión Nuclear “Guillermo Velarde”, Universidad Politécnica de Madrid, José Gutiérrez Abascal 2, E-28006 Madrid, Spain; 2 Instituto de Micro y Nanotecnología, IMN-CNM, CSIC (CEI UAM+CSIC), Isaac Newton 8, 28760 Tres Cantos, Spain; 3 Departamento de Ingeniería Energética, Escuela Técnica Superior de Ingenieros Industriales, Universidad Politécnica de Madrid, José Gutiérrez Abascal 2, E-28006 Madrid, Spain; 4 Departamento de Física, Universidad de Oviedo, E-33007 Oviedo, Spain; 5 Center of Research on Nanomaterials and Nanotechnology, CINN (CSIC—Universidad de Oviedo), El Entrego, 33940 Spain; 6 Departamento de Química Física, Universidad Complutense de Madrid, Avenida Complutense s/n, E-28040 Madrid, Spain; 7 Bionanoplasmonics Laboratory, CIC biomaGUNE, Paseo de Miramón 182, 20014 Donostia, San Sebastián Spain; 8 Centro de Láseres Ultrarrápidos, Universidad Complutense de Madrid, Avenida Complutense s/n, E-28040 Madrid, Spain

**Keywords:** Nanoscience and technology, Optics and photonics

## Abstract

One of the most important and distinctive features of plasmonic nanostructures is their ability to confine large electromagnetic fields on nanometric volumes; i.e., the so-called hot spots. The generation, control and characterization of the hot spots are fundamental for several applications, like surface-enhanced spectroscopies. In this work, we characterize the near-field distribution and enhancement of nanostructured gold thin films fabricated by glancing angle deposition magnetron sputtering. These films are composed of columnar nanostructures with high roughness and high density of inter-columnar gaps, where the electromagnetic radiation can be confined, generating hot spots. As expected, the hot spots are localized in the gaps between adjacent nanocolumns and we use scattering-type scanning near-field optical microscopy to image their distribution over the surface of the samples. The experimental results are compared with finite-difference time-domain simulations, finding an excellent agreement between them. The spectral dependence of the field-enhancement is also studied with the simulations, together with surface-enhanced Raman spectroscopy at different excitation wavelengths in the visible-NIR range, proving a broad-band response of the substrates. These findings may result in interesting applications in the field of surface-enhanced optical spectroscopies or sensing.

## Introduction

The confinement of large electromagnetic fields in the surroundings of plasmonic nanostructures has been intensively studied during the last decades due to their potential for diverse surface-enhanced applications such as surface-enhanced Raman scattering (SERS)^1–3^, surface-enhanced infrared absorption (SEIRA)^4^ or surface-enhanced fluorescence (SEF)^5,6^, as well as other applications, like catalysis^7^ or photovoltaics^8–10^. A variety of plasmonic nanomaterials are capable of sustaining localized surface plasmon resonances (LSPR) at their interface with a dielectric medium. The field confinement and enhancement produced at the surroundings of these structures depend on their shape, size, composition and dielectric medium^11,12^. Importantly, the electric field is several orders of magnitude more intense in the so-called hot spots, provided that there exist interparticle gaps in the structure and/or surface roughness^13–16^.

Several techniques are available for the fabrication of nanostructured plasmonic materials supported on substrates, such as self-assembly methods^17–21^, lithography^22,23^, or physical vapour deposition (PVD) methods. Among the latter, magnetron sputtering^24,25^ has several advantages with respect to other approaches due to its simplicity, low cost and amenability to large-scale fabrication in relatively short times. In the typical configuration, PVD is used to fabricate continuous thin films but it can also be used to produce porous nanostructured thin films, when the angle between the particle flux and the substrate normal is large enough. The nanostructures fabricated in this configuration usually have the form of nanocolumns and are the result of the so-called dynamic shadowing effect^26^. This deposition condition is usually referred to as oblique angle deposition (OAD) or glancing angle deposition (GLAD)^26–30^. Owing to the particular morphology of the resulting structures, they are able to concentrate intense electric fields in the gaps between columns. Several examples exist in the literature, mainly with silver films of nanocolumns, L-shaped structures, zig-zag structures and even nanoparticles between semiconductor nanocolumns^31–40^, whose applicability as SERS substrates has been demonstrated.

In this work we have fabricated two different types of gold nanostructured thin films by GLAD magnetron sputtering^41–43^ and characterized their optical behaviour, specifically their spatial and spectral near-field response, with a combination of finite-differences time-domain (FDTD) simulations and experimental measurements. First, the morphology and size dispersion of the nanocolumns have been assessed. Next, we have used a combination of experiments and simulations to study the correlation between the morphology and spatial distribution of the nanocolumns with the optical response of the ensemble, both in the near- and far-field. The near-field enhancement has been measured by means of scattering-type scanning near-field optical microscopy (s-SNOM), which allowed us to directly visualize the electromagnetic hot spots between the nanostructures^44–48^ and characterize their spatial distribution. The spectral response of the near-field enhancement has been determined measuring their SERS response for 532, 633 and 785 nm excitation wavelengths, which is also useful to prove their applicability as broad-band sensors.

## Results and Discussion

### Sample analysis and reflectance measurements

**Figure 1:**
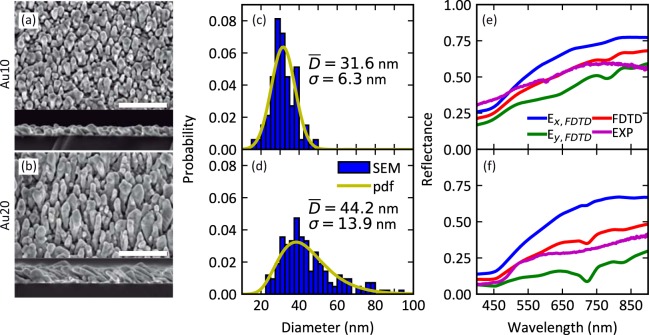
SEM images (the white scale bar is 300 nm, top view above and cross section along the particle flux direction below) of (**a**) Au10 and (**b**) Au20 samples with their corresponding (**c** and **d**) statistical analysis and (**e** and **f**) their measured and calculated reflectance. In (**c**,**d**), the blue bars represent the direct results from the analysis of the images with the corresponding mean value and standard deviation and the yellow lines show the probability distribution function (pdf) calculated with those values for a normal and log-normal distribution respectively. In (**e**,**f**), the blue and green lines denote the theoretical reflectance calculated by the FDTD method for longitudinal (*E*_*x*_) and transversal (*E*_*y*_) polarised light, the red lines represent the reflectance for unpolarised light calculated by averaging the previous values, and the purple lines correspond to the measured reflectance.

The fabricated samples (hereafter referred as Au10 and Au20, for 10 and 20 minutes of deposition time, respectively) exhibit a random distribution of tilted gold nanocolumns with high roughness and porosity, similarly to those described in previous works^41–43^. The characterization of their morphology and far-field optical response is summarized in Fig. 1 and a brief overview of their sputtering parameters and morphological features is illustrated in Table 1.

**Table 1:** Summary of the main sputtering and morphological parameters of the fabricated samples.

	Sputtering parameters				Morphological features		
	σ (°)	*P* (W)	*P*_*Ar*_ (Pa)	*T* (min)	*H* (nm)	*θ* (°)	*D* (nm)
Au10	85	100	0.15	10	50	28	36.1 ± 6,3
Au20	85	100	0.15	20	100	28	44.2 ± 13,9

The included sputtering parameters are the tilt angle of the substrate and the Au target (*σ)*, the sputtering power (*P*), the Ar pressure (*PAr*) and the deposition time (*T*). The main morphological features are the film thickness (*H*), the tilt angle between the nanocolumns and the substrate (*θ*) and the nanocolumn mean diameter and standard deviation (*D*).

The formation of nanocolumnar structures, elongated along the particle flux direction, arises from the self–shadowing effect^26–28^: the first islands, formed at the early stages of the deposition process, project a shadow behind them that obstructs the deposition of further material within these regions. To promote the self-shadowing effect, it is necessary to work in the ballistic regime, where the thermalization degree of the deposition flux is sufficiently low (in this case, Ξ ∼0.1^41,42^) to reduce the adatom diffusion compared to the Au atoms arrival time.

The samples considered in this work have a thickness (*H*) of ~50 and ~100 nm for Au10 and Au20, respectively, but the deposition time does not affect the tilt angle (*θ*) of the columns, which is ~28° for both samples^41^. More interestingly, the distribution of the nanostructures’ diameters (*D*) is fairly different in both samples, as confirmed by the statistical analysis of the top-view scanning electron microscopy (SEM) images. For sample Au10, the diameter of the columnar structures has a normal distribution and a mean value of 31.6 ± 6.3 nm, whereas sample Au20 exhibits a log-normal distribution with a mean diameter of 44.2 ± 13.9 nm and higher dispersion (blue histograms and corresponding probability density functions are depicted in Fig. 1c,d). The deposition time also affects the optical response of the samples: Au10 presents a larger reflection than Au20 (purple lines in Fig. 1e,f). However, it would be expected a larger reflectance from the sample with a larger gold content (Au20). This effect can be explained by the larger dispersion of sizes of the Au20 sample, which accounts for a larger diffuse reflectance. This phenomenon is confirmed by subsequent measurements, especially by the s-SNOM characterization.

The theoretical reflectance of the structures is also calculated by FDTD simulations (details of the FDTD method are included in the Supplementary Information, SI), using the geometrical parameters obtained from the SEM analysis (namely, the thin film thickness, tilt angle and diameter distribution of the columns) to build the computational structure. It is particularly important to apply the measured diameter distributions in the SEM analysis to generate a population of nanocolumns that accurately reproduces the optical response of the real samples. The simulations are performed in longitudinal mode, *i*.*e*., light polarisation parallel to the nanocolumns (*E*_*x*_) and in transversal mode, *i*.*e*., light polarisation perpendicular to the nanocolumns (*E*_*y*_). The reflectance is higher in the case of longitudinal polarisation for both samples. The average value of both calculations represents the reflectance for unpolarised light, which is in very good agreement with the experimental measurements, thus, validating the model for calculating the electromagnetic field enhancement. Based on the porosity, roughness, and nanostructure of the films (mainly their nanoscale size and porosity) and their low reflectance, it is evident that they present certain plasmonic behaviour that deserves further studies.

### Near-field characterization

**Figure 2:**
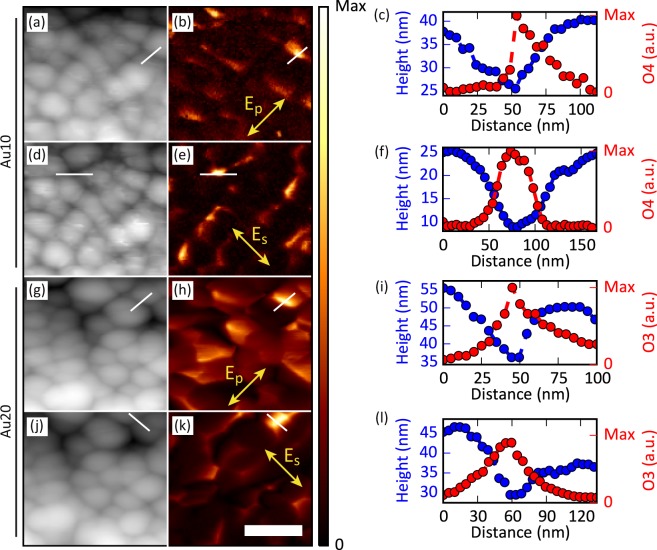
Topography (**a,d,g,j**) and s-SNOM near-field images (**b,e,h,k**) of the fabricated samples: (**a**–**f**) correspond to the Au10 sample, and (**g**–**l**) to the Au20 sample. The polarisation direction is indicated in the near-field images. Panels (**c,f,i,l**) represent the topography (blue) and near-field signal (red) extracted along the white lines in the maps next to them showing the electric field confinement in the gaps of the nanostructured films. Additional images of other areas are included in the SI. The white scale bar is 200 nm.

Characterization of the spatial distribution of electromagnetic hot spots is performed by direct imaging with an s-SNOM microscopy at an excitation wavelength of 633 nm. Fig. 2 depicts the topography of the scanned regions (500 × 500 nm^2^), together with the corresponding near-field measurements for s and p incident polarisations. The scanned region is the same for each polarisation and sample. A line scan (white lines in topography and near-field maps) along the maximum signal is also included in the images for comparison. Fig. 2a–f correspond to the Au10 sample, and Fig. 2g–l to the Au20 sample.

The plasmonic substrates clearly exhibit electromagnetic hot spots under both p- and s-polarised light and it is evident that the confinement of the electric field occurs at the gaps between the gold nanocolumns, especially at those that are perpendicular to the direction of the incident polarisation. Moreover, the represented line scans unambiguously corroborate that larger field-enhancement occurs at the gaps between the nanostructures, where the near field reaches its maximum value and the topography a minimum. The results are similar for both samples. The main difference is that sample Au20 has a higher background surface signal, for both polarisations. This can be explained by internal and diffuse reflection of the light due to the higher dispersion of size for the columns of this sample. Similar scans, performed in other regions, are included in Fig. S4.

**Figure 3:**
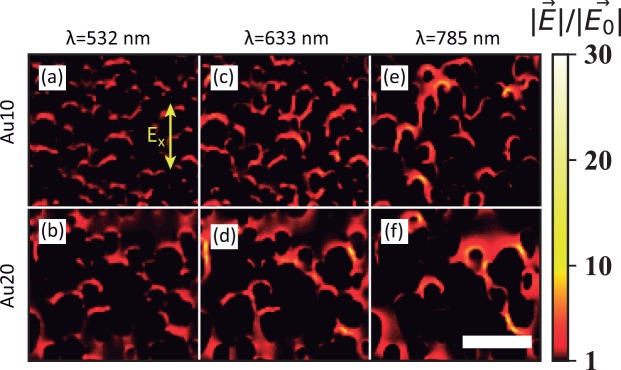
Simulated electromagnetic near-field enhancement maps at the substrate surface. Panels (**a,c,e**) show the results for the Au10 sample at wavelengths of 532, 633, and 785 nm, respectively. Panels (**b,d,f**) correspond to the Au20 sample at the same wavelengths. The incident light is polarised in the longitudinal direction of the columns (*E*_*x*_). The white scale bar is 200 nm.

**Figure 4:**
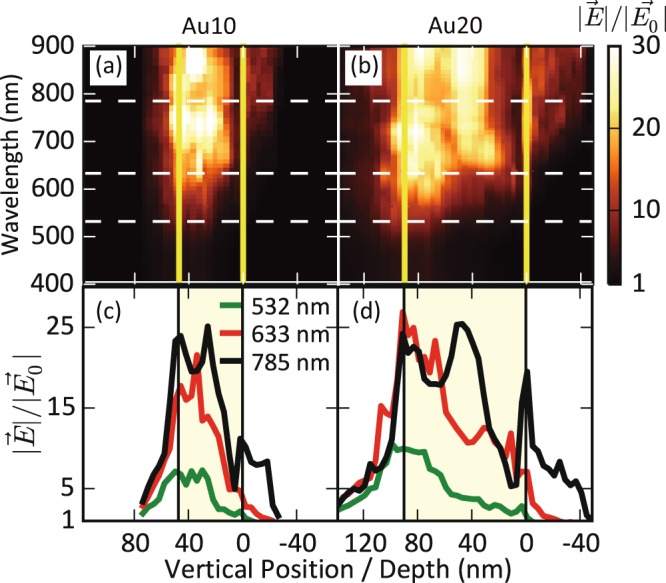
Theoretical results of the electromagnetic field-enhancement for *E*_*x*_ polarisation. Panels (**a,b**) show the maximum field-enhancement as a function of the wavelength and vertical position of the Au10 and Au20 samples respectively. Panels (**c,d**) show, respectively, the maximum field-enhancement for the Au10 and Au20 samples, as a function of the sample vertical position at 532, 633, and 785 nm, i.e., at the dashed white lines in (**a**,**b**) respectively. The origin in the vertical position scale (0 nm) denotes the interface between the nanostructured film and the substrate. The thin film is represented by the yellow vertical lines in (**a,b**) and by the pale yellow area in (**c,d**).

The theoretical calculations of the near-field enhancement (|*E*|/|*E*_0_|) are summarized in Figs. 3 and 4 for longitudinal (*E*_*x*_) polarisation. Fig. 3 shows the field-enhancement maps (500 × 500 nm^2^) at the surface for both samples at 532, 633 and 785 nm. Fig. 4 shows the maximum value of |*E*|/|*E*_0_| as a function of the incident wavelength and vertical position or depth for both samples. The results for transversal polarisation (*E*_*y*_) are included in the SI. Near-field enhancement is clearly dependent on the wavelength: larger values are always obtained for higher wavelengths, mainly above 600 nm and up to the NIR regime (Figs. 3 and 4). The value of |*E*|/|*E*_0_| is about five times smaller for wavelengths below 600 nm, compared with wavelengths above this threshold. This effect is clearly observed in the maps of the electric field. For 532 nm the field-enhancement lies in the range 1–5 but for 633 and 785 nm enhancements of up to 30 are achieved. This is due to the coupling of the LSPR of the individual columns, which broadens and redshifts the LSPR band^49,50^. The LSPR coupling is also responsible for the similar response observed for transversal polarisation (Figs. S2 and S3).

Light polarisation also affects the spatial distribution of the near-field enhancement. Ideally, the longitudinal polarisation increases the field at the tip of a single Au nanorod whereas the transversal polarisation increases the fields on its sides. The same trend is observed in these samples for both polarisations (Figs. 3 and S2), with some exceptions, due to the proximity of the individual structures. These theoretical results are also in good agreement with the near-field distribution and with the polarisation dependence observed in the s-SNOM near-field images. The near-field enhancement is also attenuated with the vertical position or depth of the samples and its maximum value located in the sample surface. This effect can be clearly appreciated in Fig. 4, remarking their applicability for surface-enhanced applications, where the probe molecule must be adsorbed at the substrate surface.

**Figure 5:**
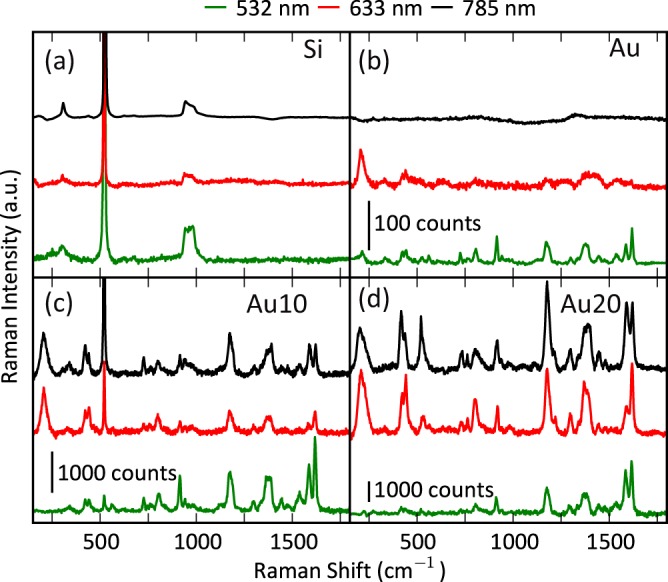
Background-subtracted CV SERS spectra (1 µM) of (**a**) bare Si, (**b**) Au thin film grown in normal configuration, (**c**) Au10 and (**d**) Au20 samples measured at excitation wavelengths of 532 (0.14 mW), 633 (0.08 mW) and 785 nm (2.35 mW).

In order to evaluate the performance of both samples in the visible-NIR wavelength range, we measured the SERS spectra of crystal violet (CV) using excitation wavelengths of 532, 633 and 785 nm. All the spectra correspond to samples incubated in 1 µM of CV aqueous solution. The background-subtracted SERS spectra are shown in Fig. 5, together with the same measurements in incubated bare Si substrates and gold substrates grown by normal configuration. No Raman signal was detected in the silicon substrates. Regarding the gold substrates grown with normal configuration, a very low Raman signal is detected in the visible wavelength (i.e., not for 785 nm) whereas the SERS spectra can be detected in both columnar samples. Regarding the gold content, higher SERS signals are observed for sample Au20 but its background fluorescence is also larger (raw spectra in Fig. S5), which might outstrip the Raman peaks. This is an indication that the samples behave as broadband plasmonic sensors capable of performing well in the visible-NIR range. However, the Raman peaks are better resolved at longer wavelengths. This effect might be explained by the red-shift of the near-field enhancement, because the crystal violet differential Raman cross section and absorbance have a resonance between 532 and 650 nm^51^. However, this is hard to assert, due to the presence of the enhanced fluorescence that accompanies the SERS signals and also depends on the proximity of the molecule to the metal surface. This proximity affects the radiative and non-radiative decay processes and, therefore, the SERS spectra at 532 nm also suggest a partial quenching of the fluorescence^52–56^ that suppresses part of the Raman features. Moreover, the analytical enhancement factor ((I_SERS_/c_SERS_)/(I_RS_/c_RS_))^52^ for each wavelength is estimated with reference to a 40 μL of 5 mM CV solution dried on a bare Si substrate of about 1 × 1 cm^2^. The peaks at 1590 and 1620 cm^−1^ have been used for this task. The estimated values for 532 and 633 nm are in the range of 10^3^ and 10^4^ for Au10 and Au20 samples, respectively. For 785 nm, the results are in the range of 10^2^ and 10^3^ for Au10 and Au20 samples, respectively. These values are only an estimation due to the difficulty calculating the actual number of molecules that are excited. On one hand, the molecules will be dragged to the inter-columnar gaps of the nanostructured films due to the surface tension and, on the other hand, the reference non-SERS spectra were measured in the outwards of the dried solution and, in all likelihood, the actual concentration of molecules is higher than the nominal. The variations in the relative peak intensities of the SERS spectra at different excitation wavelength arises from several reasons: local variations of the SERS substrate, presence of enhanced fluorescence and the excitation wavelength in resonant or non-resonant conditions. For crystal violet, the wavelengths of 532 and 633 nm excite resonantly whereas the wavelength of 785 nm produces a non-resonant excitation^51^, and the variation of the relative peak intensities is related to the charge-transfer in resonant or non-resonant conditions, which means that the vibrations are selectively enhanced according with the Herzberg-Teller-surface selection rules^57–60^.

**Figure 6:**
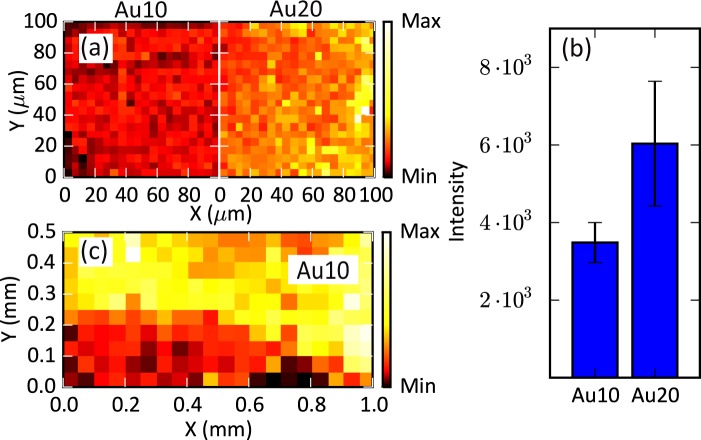
Background-subtracted SERS spectra maps of the intensity at ∼205 cm^−1^ mode of CV measured at an excitation wavelength of 633 nm: 100 × 100 μm^2^ maps (**a**) with a spatial resolution of 5 μm and their corresponding histograms (**b**) and 1 × 0.5 mm^2^ map **(c**) with a spatial resolution of 50 μm.

Nevertheless, the main advantage of this kind of substrates comes from its surface homogeneity at the large scale. To show this feature, Fig. 6 represents the SERS intensity maps of several areas measured with the 633 nm excitation wavelength. This figure includes the mapping of a 100 × 100 μm^2^ area for both samples, with a spatial resolution of 5 μm, and the statistical analysis of the 400 measured spectra of each map. The Au10 sample shows a mean intensity of about 3500 counts with a standard deviation of 15% whereas the Au20 sample exhibits a mean intensity of 6000 counts with a standard deviation of 25%. Additionally, for the Au10, a larger map (separated several mm away from the former) with a size of 1 × 0.5 mm^2^ and a spatial resolution of 50 μm is included. The main drawback of performing such large maps is the possible loss of focus that can affect the measured signal. In any case, the recorded spectra exhibit good quality and homogeneity along the full area. The combination of both maps demonstrates the uniformity of the samples’ surface at the macroscopic range. This fact, together with the possibility of deposit large areas with short fabrication time, characteristic of the sputtering process, gives a high versatility and ease of use to this kind of substrates.

Finally, we can compare the SERS performance of our nanocolumnar samples with that of similar substrates grown either by the same technique or by other methods, such as lithography. The main difference between the lithography-made substrates^61–67^, and GLAD substrates is that the former exhibit an ordered structure, whereas in the latter the columns are randomly located. Ordered structures may be advantageous to tailor the absorption and increase the substrate sensitivity at certain wavelengths. However, considering the excitation power, analyte concentration, integration time and final SERS intensity (used laser powers can range from 0.1 to 100 mW, integration time from 1 to 10 seconds usually and estimated enhancement factors from 10^3^ to 10^9^)^61–67^ revealed that our samples can behave similarly to the average lithography substrate. Furthermore, our samples have several advantages, stemming from the fabrication method, because GLAD with magnetron sputtering is a scalable technique that allows manufacturing at large scale, with low-cost and short fabrication times. The most important fact is the high macroscale uniformity of the substrate at large areas. Moreover, the fabrication is very simple, in comparison to the complexity and little scalability required for the more expensive lithography methods, which also require the use of several chemicals, such as resists. Finally, it should be mentioned that, if the application demands it, structures with higher sensitivity can be grown by GLAD, for instance, by cooling down the substrate to temperatures around −140 °C^68,69^, but at the expense of complicating the fabrication method.

## Conclusions

In this work we have fabricated nanostructured gold substrates by glancing angle deposition magnetron sputtering. Moreover, we have studied their morphology and behaviour in the near-field by a combination of experimental measurements (SERS and s-SNOM) and FDTD simulations, observing good agreement between them. It is shown that these substrates are useful for broadband sensing applications with good sensitivity, like that obtained with substrates fabricated with more expensive and complicated methods, such as lithography. The present fabrication method is ideal for the commercial production of SERS substrates due to its low-cost, large-scale amenability, and short time required for manufacturing, particularly if we compare it with complex multi-step fabrication and expensive lithography techniques. This work may open the door to the application of SERS in areas sensitive to the cost–performance relation, such as chemical or biological sensing for medical applications.

## Methods

### Sample fabrication

**Figure 7:**
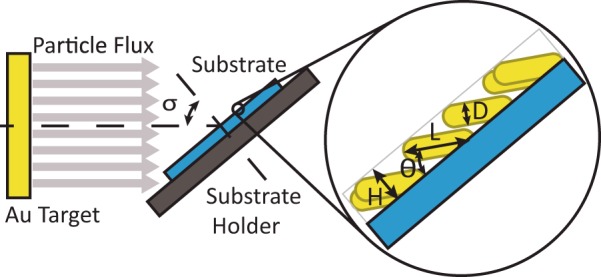
Schematic representation of the experimental setup for the growth of gold nanostructured porous thin films by GLAD magnetron sputtering, where σ is the tilt angle between the Si substrate and the normal direction to the Au target. Nanocolumns longitudinal (L) and transversal (D) dimensions are depicted in the inset along with the angle θ between the longitudinal dimension of the nanocolumns and the Si substrate and the distance (*H)* between the outer edge of the nanoparticles and the Si substrate (film thickness).

The gold nanostructured samples were fabricated on 1 × 1 cm^2^ Si (100) substrates by DC magnetron sputtering at room temperature (RT) in an ultra-high vacuum (UHV) chamber (base pressure in the low 10^−9^ mbar range). The target diameter was 3.8 cm and the distance between the target and substrate was 19 cm. Before their introduction in the chamber, the Si substrates were ultrasonically cleaned in acetone. In order to obtain columnar nanostructures, the glancing angle deposition (GLAD) method was employed^27^, with a tilt angle (σ) between the target and substrate of 85°. The applied deposition times were 10 and 20 min. The power was set to 100 W and, in favour of the ballistic regime and to promote the shadowing effects that lead to the formation of nanostructures^41,42^, the Ar pressure was set to the lowest value that gives a stable plasma (0.15 Pa). A 3 nm thick Ti buffer layer was firstly deposited using the standard configuration. *i*.*e*. with no tilt angle, to increase the adhesion between the Si substrate and the gold nanostructured layer. Fig. 7 depicts a schematic representation of the experimental setup.

### Morphology characterization

The morphology of the fabricated structures was characterized by Scanning Electron Microscopy (SEM) using a FEI Verios 460 Field Emission setup. The ImageJ^70^ software was employed for the analysis of the main geometrical parameters of the samples, *i*.*e*., the angle of the nanocolumns relative to the substrate (*θ*), their length (*L*), and diameter (*D*), as well as the film thickness (*H*). About 500 nanocolumns were measured per sample.

### Reflectance measurements

The reflectance of the samples was measured using a custom-made optical microscope. The unpolarised white light from a halogen lamp (Ocean Optics D-H 2000) was guided by a silica optical fibre with a 200 μm diameter core and converted to a nearly parallel beam using a silica lens. The parallel beam was directed with a 50:50 beam splitter to a 10x objective and focused on the sample. The reflected light was collected with the same objective, through the beam splitter, and then focused with another silica lens into a silica optical fibre with a 1 mm diameter core. The light was guided to a compact spectrometer (Ocean Optics USB 2000+) configured with a multichannel array detector to simultaneously measure the spectrum in the range of 400–900 nm, with a spectral resolution better than 1 nm. Reflectance spectra were recorded using an integration time of 10 seconds.

### SERS measurements

The substrates were incubated in ~1 mL of a 1 μM aqueous crystal violet (CV, dye content greater than 90% and purchased from Sigma-Aldrich) solution for 15 minutes. The substrates were dried in air several hours before recording the SERS spectra. SERS measurements were carried out in a confocal Raman microscope (Renishaw inVia Reflex) using an excitation wavelength of 532, 633 and 785 nm lasers as excitation sources. A 1800 grooves/mm diffraction grating was used for 532 and 633 nm laser lines and a 1200 grooves/mm diffraction grating for the 785 laser line. In all cases a front-illuminated Peltier-cooled CCD camera with 1024 × 512 pixels was used. SERS spectra were recorded using a 50x objective with variable laser power and an integration time of 10 seconds in extended mode to measure the same spectral range (150–1800 cm^−1^) for all the excitation wavelengths. Each spectrum was measured at different points of the samples to check their homogeneity. The SERS spectra for the maps were recorded with an integration time of 2 seconds and a laser power of about 1 mW at an excitation wavelength of 633 nm.

### Near-Field measurements

**Figure 8:**
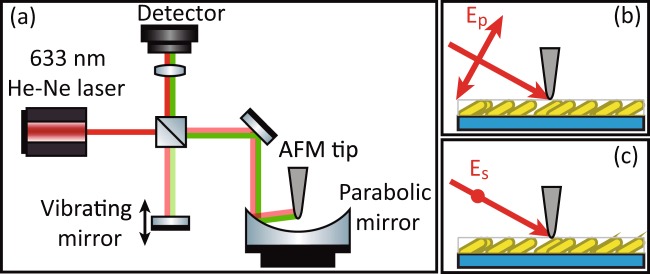
(**a**) Schematic representation of the s-SNOM experimental setup for the simultaneous measurement of sample topography and near-fields, and (**b**,**c**) incidence of the incoming light with p- and s- polarisations, respectively.

Direct visualization of the electromagnetic hot spots over the surface of the samples, together with their topography, was performed using a scattering-type scanning near-field optical microscope (s-SNOM, Neaspec GmbH). In this equipment, a coated (Pt/Ir) AFM tip oscillating at its resonant frequency (*Ω* ≈ 285 kHz) with a tapping amplitude of about 50 nm is illuminated with a He-Ne laser (633 nm) while it raster scans the plasmonic substrate. The back-scattered near-fields are imaged by recording the tip-scattered field with a pseudo-heterodyne Michelson interferometer as a function of tip position, yielding near-field images (O) that are demodulated at the harmonic frequency of the tip vibration frequency *nΩ* (*n* ≥ 3). The measurements were performed twice, *i*.*e*., for incident p- and s-polarised light. Under p-polarised incident light, a strong dipolar interaction between tip and sample will also be present due to the vertical component of the electric field, an effect that is dramatically reduced with the s-polarised light. Hence, the main difference between both polarisations is that the measurements under p-polarised light present a strong near-field contribution from the surface material (Au) together with that of the excited hot spots, while under s-polarised light mainly the near-field contribution from the hot spots is measured. A schematic representation of the experimental s-SNOM setup is depicted in Fig. 8.

## Supplementary information

Supplementary info

## Data Availability

The data generated and analysed during the current study will be made available from the corresponding author on reasonable request.
