# Supplementary material for: On the Large Near-Field Enhancement on Nanocolumnar Gold Substrates
Source: Sci Rep. 2019 Sep 26;9:13933. doi: 10.1038/s41598-019-50392-w (PMC6763449; doi:10.1038/s41598-019-50392-w)
Supplement: Supplementary file 1 — Supplementary info [file 41598_2019_50392_MOESM1_ESM.pdf]

Supplementary Information

On the Large Near-Field Enhancement on Nanocolumnar Gold Substrates

Pablo Díaz-Núñez[1,*], José Miguel García-Martín[2], María Ujué González[2], Raquel González-Arrabal[1,3], Antonio Rivera[1,3], Pablo Alonso-González[4,5], Javier Martín-Sánchez[4,5], Javier Taboada-Gutiérrez[4,5], Guillermo González-Rubio[6,7], Andrés Guerrero-Martínez[6], Luis Bañares[6,8] and Ovidio Peña-Rodríguez[1,3]

[1]Instituto de Fusión Nuclear "Guillermo Velarde", Universidad Politécnica de Madrid, José Gutiérrez Abascal 2, E-28006 Madrid, Spain.
[2]Instituto de Micro y Nanotecnología, IMN-CNM, CSIC (CEI UAM+CSIC), Isaac Newton 8, 28760 Tres Cantos, Spain.
[3]Departamento de Ingeniería Energética, Escuela Técnica Superior de Ingenieros Industriales, Universidad Politécnica de Madrid, José Gutiérrez Abascal 2, E-28006 Madrid, Spain.
[4]Departamento de Física, Universidad de Oviedo, E-33007 Oviedo, Spain.
[5]Center of Research on Nanomaterials and Nanotechnology, CINN (CSIC—Universidad de Oviedo), El Entrego 33940, Spain
[6]Departamento de Química Física, Universidad Complutense de Madrid, Avenida Complutense s/n, E-28040 Madrid, Spain.
[7]Bionanoplasmonics Laboratory, CIC biomaGUNE, Paseo de Miramón 182, 20014 Donostia-San Sebastián, Spain.
[8]Centro de Láseres Ultrarrápidos, Universidad Complutense de Madrid, Avenida Complutense s/n, E-28040 Madrid, Spain.
*p.diazn@alumnos.upm.es

Contents

1. FDTD simulation details

2. FDTD results for transversal (E_y) polarization

3. Additional s-SNOM measurements in other areas of the samples

4. Raw SERS spectra of crystal violet as a function of laser power

References

1. FDTD simulation details

The optical response of the structures is studied theoretically by means of FDTD simulations, performed with the free computer code MEEP[1]. A full description of the FDTD method can be found elsewhere.[2,3] Fig. S1 shows the implemented simulation cell. The structure is constructed as randomly distributed hemispherically capped cylinders aligned with the X-axis over a substrate, each one representing a nanocolumn. Two structures are simulated, each one representing one of the fabricated samples. The angle of the nanocolumns with the substrate (θ) is set to 28° for both samples and their length (L) is set to ~100 nm for the sample at 10 minutes of deposition time and ~200 nm for the sample at 20 minutes. The angle and length are considered constant for all the columns. However, their diameter (D) varies following the statistical distribution calculated from the SEM images. The simulation cell has a size of $10^3 \times 10^3 \times 7H$ nm^3 with Bloch-periodic translational symmetry in the X and Y directions and perfectly matched layers (PMLs, grey areas in Fig. S1) of thickness 2H in the Z direction to absorb the reflected and transmitted waves, where H denotes the thickness of the nanostructured film. The columnar structure is placed at the center of the simulation box. A broadband Gaussian source (400-900 nm) is placed at the top of the cell and the electromagnetic wave propagates along the Z direction. The simulation is performed for polarized light along the "X" axis (E_x) and polarized light along the "Y" axis (E_y). The fields are allowed to evolve and the simulation is terminated once the ratio $|\mathbf{E}|^2/|\mathbf{E}_{max}|^2$ has decayed down to 10^{-6} at the bottom of the cell (black point in Fig. S1).

The optical response in the far field is analyzed by means of the reflectance (R), which is calculated as the quotient between the reflected power by the structure (W_R) and the incident radiation (W_I) in a non-absorbing medium: $R = W_R/W_I$. The values of W_R and W_I are calculated as the integral of the Poynting vector of the Fourier-transformed electric and magnetic fields at each frequency (ω) over an area above the structure (upper red dashed line in Fig. S1) and below the structure (lower red dashed line in Fig. S1) respectively. The incident radiation is calculated without the structure and the incident fields are subtracted from the reflected fields in the reflectance calculations.

$$W_I = \Re \iint E^*_{\omega,0} \times H_{\omega,0} dS \qquad (1)$$

$$W_R = \Re \iint (E_\omega - E_{\omega,0})^* \times (H_\omega - H_{\omega,0}) dS \qquad (2)$$

Finally, the near-field response is evaluated by means of the field enhancement, $|\mathbf{E}|/|\mathbf{E}_0|$. Each component of the complex electric field (E_x, E_y and E_z) is stored at each time step and every point of the space in the simulation cell. Once the fields, in the form of $\mathbf{E} = \mathbf{E}(x, y, z, t)$, are accumulated over the full simulation time, they are Fourier-transformed to obtain a full spectrum of the electric

fields in the frequency domain, **E** = **E**(x, y, z, ω), for all the space considered in the simulation cell. These calculations are performed twice: first with the structure to obtain the scattered field (|**E**|) and then without the structure to obtain the incident field (|$\mathbf{E}_0$|) so as to normalize the former data.

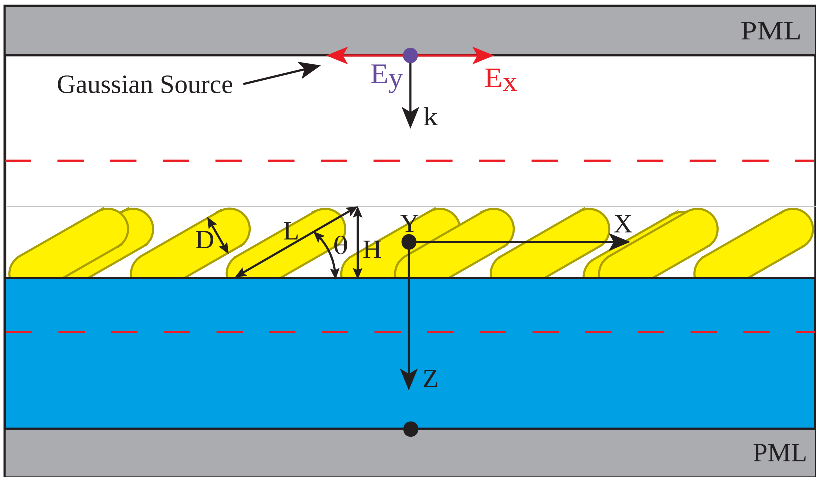

Figure S1: Schematic representation of the simulation cell used in the FDTD calculations. The grey areas represent the PMLs, used to absorb the reflected and transmitted waves. The dashed red lines represent the areas where the integral of the Poynting vector was applied to calculate the reflected, transmitted and incident power. The simulations were performed for longitudinal (red) and transversal (blue) polarized light propagating along the Z direction. The fields were monitored at the black point at the bottom of the cell to stop the simulation once they had sufficiently decayed.

2. FDTD results for transversal (E_y) polarization

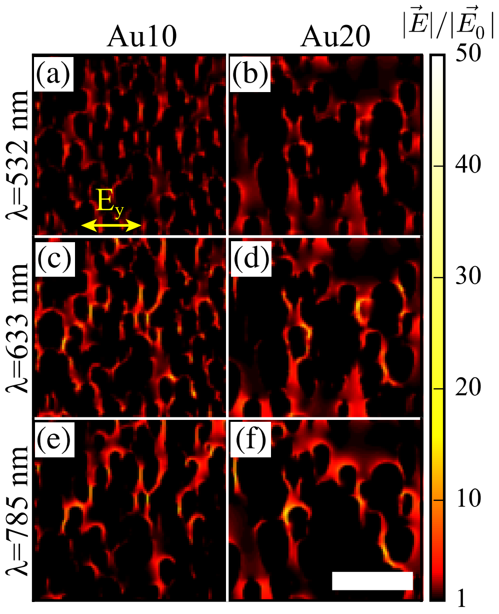

Figure S2: Simulated electromagnetic near-field enhancement maps at the substrate surface. Panels (a), (c), and (e) show the results for the Au10 sample at wavelengths of 532, 633, and 785 nm, respectively. Panels (b), (d), and (f) correspond to the Au20 sample at the same wavelengths. The incident light is polarized in the perpendicular direction of the columns (E_y). The white scale bar is 200 nm.

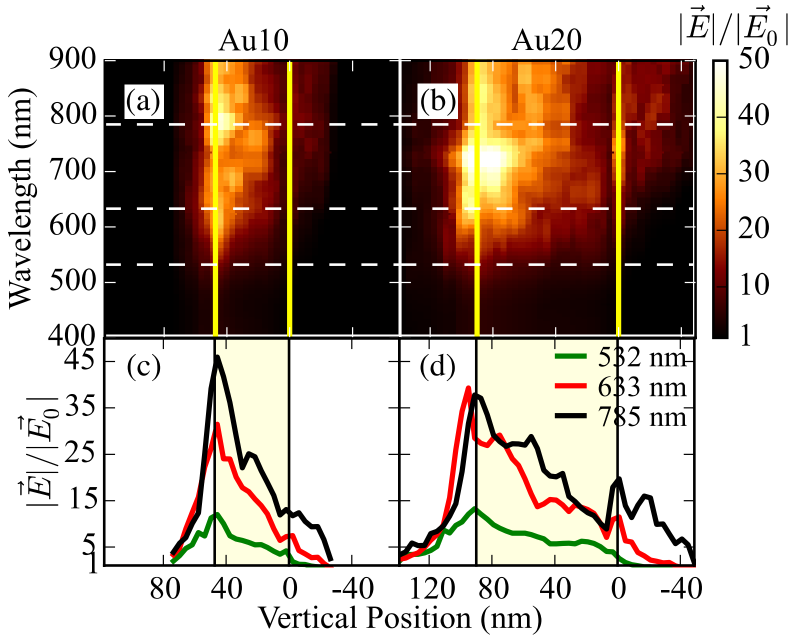

Figure S3: Theoretical results of the electromagnetic field-enhancement for E_y polarization. Panels (a) and (b) show the maximum field-enhancement as a function of the wavelength and vertical position of the Au10 and Au20 samples respectively. Panels (c) and (d) show, respectively, the maximum field-enhancement for the Au10 and Au20 samples, as a function of the sample vertical position at 532, 633, and 785 nm, *i.e.*, at the dashed white lines in (a) and (b) respectively. The origin in the vertical position scale (0 nm) denotes the interface between the nanostructured film and the substrate. The thin film is represented by the yellow vertical lines in (a) and (b) and by the pale yellow area in (c) and (d).

3. Additional s-SNOM measurements in other areas of the samples

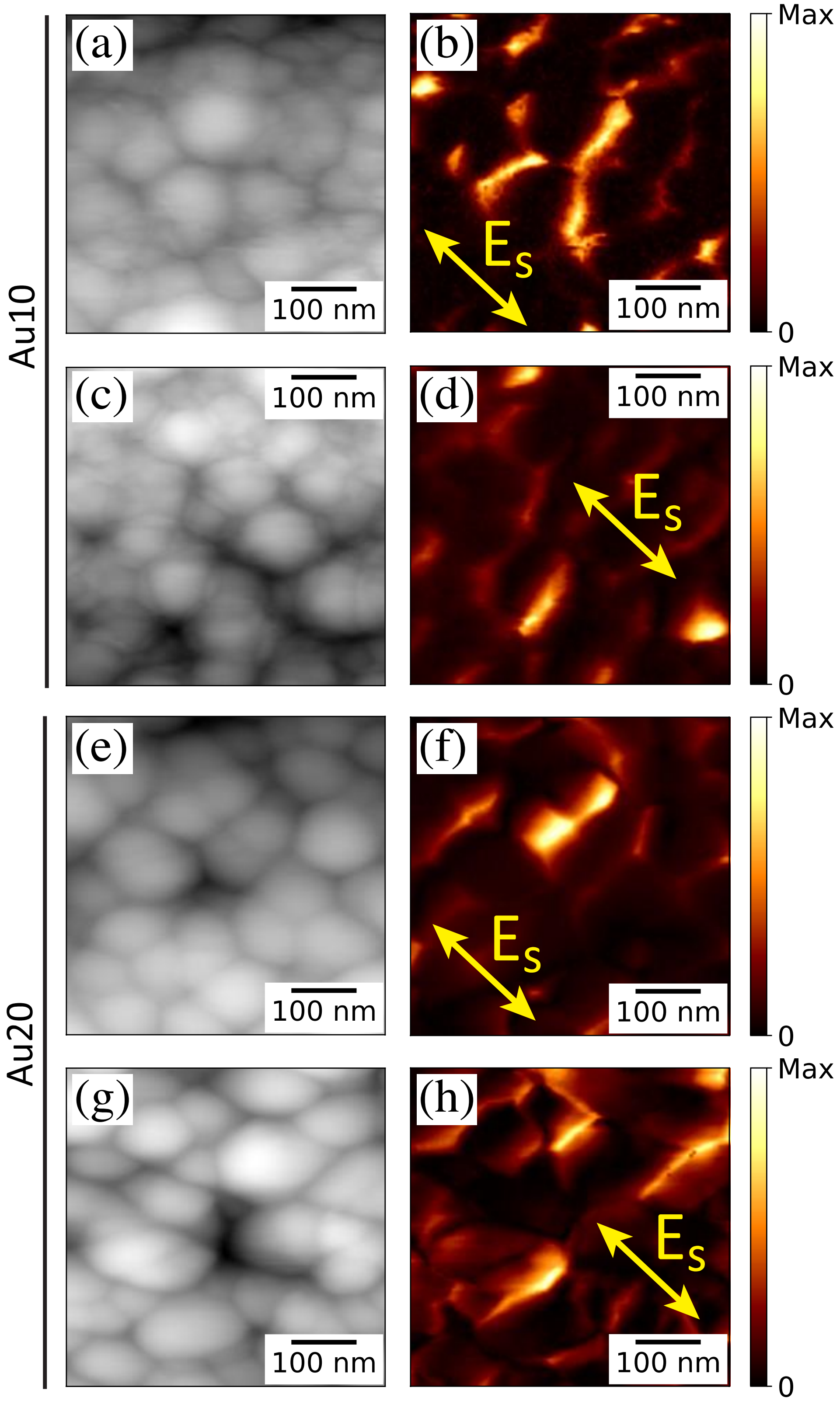

Figure S4: Additional s-SNOM topography (left) and near-field (right) maps of both samples measured at the fourth harmonic.

4. Raw SERS spectra of crystal violet as a function of laser power

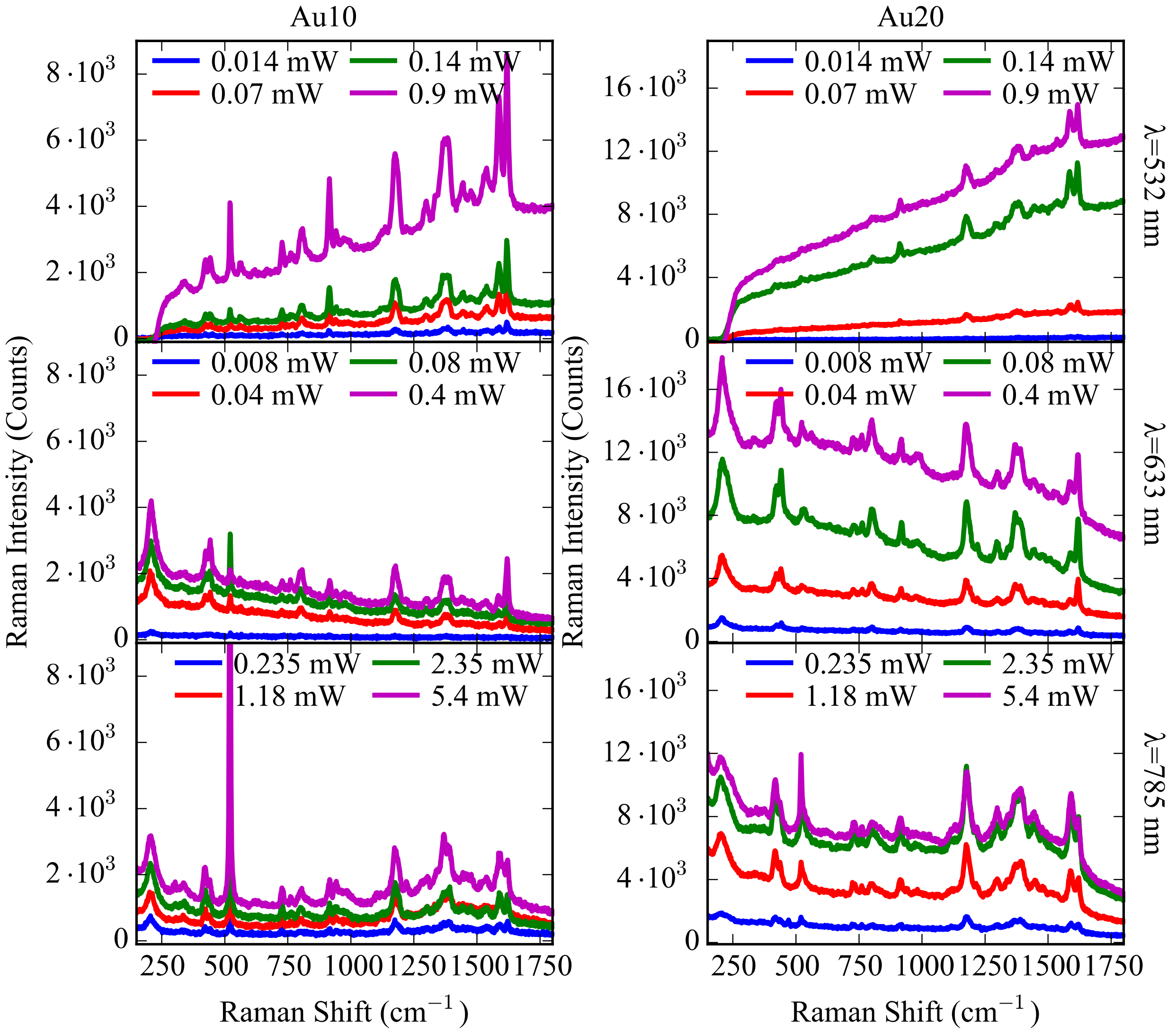

Figure S5: SERS spectra of Crystal Violet (1 µM, without background subtraction) for the Au10 (left) and Au20 (right) samples. The excitation wavelengths are 532 (upper panels), 633 (middle panels) and 785 nm (lower panels). Legend values indicate the applied laser power.

References

1. Oskooi, A. F. *et al.* Meep: A flexible free-software package for electromagnetic simulations by the FDTD method. *Comput. Phys. Commun.* 181, 687–702 (2010).
2. Taflove, A. & Hagness, S. C. *Computational Electrodynamics: The Finite-Difference Time-Domain Method.* (Artech House, 2005).
3. Taflove, A., Oskooi, A. F. & Johnson, S. G. *Advances in FDTD Computational Electrodynamics: Photonics and Nanotechnology*. (Artech House, 2013).
